# Hippocampal BAIAP2 prevents chronic mild stress-induced depression-like behaviors in mice

**DOI:** 10.3389/fpsyt.2023.1192379

**Published:** 2023-05-10

**Authors:** Yaling Fu, Xiangfei Guo, Rui Yang, Hao Feng, Xueyong Yin, Shuang Wang, Li Song, Xi Wang, Penghui Zhao, Sheng Wang, Yun Shi, Haishui Shi

**Affiliations:** ^1^Neuroscience Research Center, Institute of Medical and Health Science, Hebei Medical University, Shijiazhuang, China; ^2^Hebei Key Laboratory of Neurophysiology, Shijiazhuang, China; ^3^Department of Biochemistry and Molecular Biology, Hebei Medical University, Shijiazhuang, China

**Keywords:** chronic mild stress (CMS), depression, hippocampus, mice, brain-specific angiogenesis inhibitor 1-associated protein 2, spine density

## Abstract

**Background:**

The pathogenesis of depression is closely related to changes in hippocampal synaptic plasticity; however, the underlying mechanism is still unclear. Brain-specific angiogenesis inhibitor 1-associated protein 2 (BAIAP2), a postsynaptic scaffold protein in excitatory synapses important for synaptic plasticity, is highly expressed in the hippocampus and has been implicated in several psychiatric disorders. However, the role of BAIAP2 in depression remains poorly understood.

**Methods:**

In the present study, a mouse model of depression was established via exposure to chronic mild stress (CMS). An adeno-associated virus (AAV) vector expressing BAIAP2 was injected into the hippocampal brain region of mice and a BAIAP2 overexpression plasmid was transfected into HT22 cells to upregulate BAIAP2 expression. Depression- and anxiety-like behaviors and dendritic spine density were examined in mice using behavioral tests and Golgi staining, respectively. *In vitro*, hippocampal HT22 cells were treated with corticosterone (CORT) to simulate the stress state, and the effect of BAIAP2 on CORT-induced cell injury was explored. Reverse transcription-quantitative PCR and western blotting were employed to determine the expression levels of BAIAP2 and those of the synaptic plasticity-related proteins glutamate receptor ionotropic, AMPA 1 (GluA1), and synapsin 1 (SYN1).

**Results:**

Mice exposed to CMS exhibited depression- and anxiety-like behaviors accompanied by decreased levels of BAIAP2 in the hippocampus. *In vitro*, the overexpression of BAIAP2 increased the survival rate of CORT-treated HT22 cells and upregulated the expression of GluA1 and SYN1. Consistent with the *in vitro* data, the AAV-mediated overexpression of BAIAP2 in the hippocampus of mice significantly inhibited CMS-induced depression-like behavior, concomitant with increases in dendritic spine density and the expression of GluA1 and SYN1 in hippocampal regions.

**Conclusion:**

Our findings indicate that hippocampal BAIAP2 can prevent stress-induced depression-like behavior and may be a promising target for the treatment of depression or other stress-related diseases.

## 1. Introduction

Depression is a common neuropsychiatric disease characterized by a lack of interest, depressed mood, psychomotor retardation, and changes in daily activity behaviors (1). The global prevalence of mental disorders is estimated at 13%, with depression and anxiety disorders accounting for 60.7% of cases (2). Depression is associated with self-harm, including a high rate of suicide, and represents a significant social and economic burden (3, 4). Despite this, the causes and underlying mechanisms of depression remain poorly understood, and novel approaches for preventing or treating the disease are urgently needed.

Stress can be described as any environmental factor that disrupts physiological and biochemical homeostasis and affects internal balance. Chronic stress is a major risk factor for the development of depression (5, 6). Many studies have shown that the central nervous system of patients with depression undergoes structural and functional changes, particularly a reduction in the levels of synaptic plasticity-related proteins and the loss of dendritic spines in hippocampal brain regions (7–11). We and others have shown that exposure to chronic mild stress (CMS) can decrease the density and complexity of dendritic spines in the murine hippocampus (12–16). These observations underscore the high sensitivity of the hippocampus to chronic stress.

Brain-specific angiogenesis inhibitor 1-associated protein 2 (BAIAP2) is a postsynaptic density scaffolding protein that is highly expressed in the central nervous system, especially in the hippocampus (17–20). BAIAP2 is an important regulator of actin-rich dendritic spines and an essential component of the postsynaptic densities of excitatory synapses. The abnormal expression or function of BAIAP2 is closely associated with autism, attention-deficit/hyperactivity disorder, and schizophrenia (21, 22). Despite studies showing that BAIAP2 has a neuroprotective function, it is unknown whether this protein in the hippocampus has an impact on depression.

In the present study, we explored the role of hippocampal BAIAP2 in the pathophysiology of depression. Specifically, we investigated whether BAIAP2 expression is altered in mice subjected to CMS, assessed the effect of BAIAP2 on corticosterone (CORT)-induced injury in HT22 cells, and investigated whether BAIAP2 can block CMS-induced depression-like behavior in mice. Our findings shed light on the role of BAIAP2 in depression and highlight the therapeutic potential of targeting hippocampal BAIAP2 for the treatment of this condition.

## 2. Materials and methods

### 2.1. Animals

Male ICR mice, 7 weeks old and of similar body weight, were obtained from Beijing Vital River Laboratory Animal Technology Co, Ltd. Mice were maintained under standard conditions (temperature: 22–23°C; relative humidity: ∼60%; 12-h light, 12-h dark cycle) with *ad libitum* access to food and water. All behavioral experiments involving mice were performed during the dark period.

The trial was approved by the Laboratory Animal Ethical and Welfare Committee of Hebei Medical University.

### 2.2. Cell culture and treatment

HT22, an immortalized mouse hippocampal neuronal cell line, was purchased from Bluefbio Biology Technology Development Co., Ltd (cat. no. BNF60808571, Shanghai, China), and incubated in high-glucose DMEM containing 10% FBS and 1% penicillin–streptomycin–amphotericin B at 37°C with 5% CO_2_. To simulate the stress state *in vitro*, HT22 cells were pretreated with 200 μM corticosterone (CORT, cat. no. B7469, APExBIO, Houston, TX, USA) for 24 h.

### 2.3. Cell viability assays

A Cell Counting Kit-8 (CCK-8, cat. no. K009, ZETA, USA) was used to test the viability of HT22 cells. Cells (1 × 10^4^ per well) were seeded in 96-well plates and incubated for 24 h at 37°C. Before the assessment of viability, the cells were washed with phosphate-buffered saline (PBS), and then treated with 10 μL of CCK-8 solution in 100 μL of DMEM per well for 1.5 h. The absorbance of each well at 450 nm was recorded using a SpectraMax Absorbance Reader (Molecular Devices, China).

### 2.4. Plasmid transfection

HT22 cells were seeded in 6-well plates and, the next day, were transfected with GV388-BAIAP2 (NM_001037755), a BAIAP2 expression plasmid, or GV388-CON, the control plasmid. The plasmids were synthesized by Shanghai Genechem Co., Ltd (Shanghai, China). The cells were transfected with 4 μg of either plasmid using Lipofectamine-2000 transfection reagent (5 μL/well; cat. no. 11668-019, Invitrogen, CA, USA) according to the manufacturer’s instructions.

### 2.5. CMS

After 5 days of acclimation, ICR mice were individually housed and subjected to CMS. The model of depression was established as previously described (5, 23). The animals received two or three different stressors each day, administered on a random schedule, for 4 consecutive weeks. The stresses included damp bedding for 24 h, crowding for 24 h, cage tilting at a 60° angle for 24 h, tail clamping for 2 min, cold stimulation for 5 min, empty cage for 24 h, light/dark cycle reversal for 24 h, food deprivation for 12 h, restraint for 2 h, and water deprivation for 12 h. Control mice were kept in their home cages under normal conditions and were not subjected to stressors (23).

### 2.6. Open field test (OFT)

The OFT was used to assess locomotor activity, anxiety-like behavior, and exploratory behavior based on previous studies (24, 25). The OFT was performed in a black box (40 cm × 40 cm × 40 cm) and a 10 cm × 10 cm zone in the center of the box was defined as the center square. At the start of each test, a mouse was placed in the central zone and allowed to freely explore the box for 5 min. Mice were tracked and the results were analyzed using an automated tracking system (SMART v3.0.02, Pan Lab, Harvard Apparatus, MA, USA). The total distance traveled was recorded to evaluate the general locomotor activity of each mouse. The time spent in the central area was used to estimate anxiety-like behavior (26).

### 2.7. Novelty-suppressed feeding (NSF) test

The NSF test serves as a measure of anxiety-like behavior in mice (27). The day after being deprived of food, mice were placed in the corner of the test box (40 cm × 40 cm × 40 cm) in which a single pellet of food had been placed in the central region. The mouse was allowed 5 min to eat the pellet. Latency to feeding was recorded as 5 min if the mouse did not eat or the time to the first bite if the mouse ate (28).

### 2.8. Sucrose preference test (SPT)

The SPT was used to assess the loss of pleasure, a core symptom of depression, and was performed as previously described (27). Mice were given two bottles of a 1% sucrose solution 48 h before the experiment. The animals were then single-housed, fasted, and deprived of water for 24 h. Subsequently, the mice were given free access to either a 1% sucrose solution or water. Water and 1% sucrose solution consumption was calculated after 24 h.

### 2.9. Forced swimming test (FST)

The FST was used to assess depressive-like behavior and was performed as previously described (25). For the test, mice were individually placed in a transparent glass cylinder (45 cm in height, 20 cm in diameter) containing water (25 ± 2°C, 20 cm deep) for a total of 6 min. Total immobility time and latency to the first immobility were measured (29).

### 2.10. Novel object recognition (NOR) test

The NOR test was used to evaluate the learning and memory ability of the mice (30). The equipment for the test consisted of a black, 40-cm-wide × 40-cm-long × 35-cm-high box with an open top. For acclimation, mice were placed in the open field and were allowed to freely explore for 5 min, once a day for 3 days. The training period took place within 24 h after acclimation, during which mice were allowed to explore two identical objects in the box for 5 min. The recognition index (%) was calculated using the following formula: time spent on one object/(total interaction time with two objects) × 100%. During the test stage, one of the two identical objects was replaced with a novel object. Mice were again placed in the to explore for 5 min. The total exploration time of the familiar and novel objects was recorded. The recognition index (%) was calculated using the following formula: interaction time with the novel object/(total interaction time with two objects) × 100%.

### 2.11. Intracerebral stereotaxic injection

The mouse Bai*ap2* gene was cloned into GV388 vector (pCMV bGlobin-MCS-EGFP-hGH polyA), which was then packaged into adeno associated viruses 9 (AAV-BAIAP2) by Genechem (Shanghai, China). Mice were anesthetized with pentobarbital sodium (50 mg/kg, intraperitoneal injection) and secured in a stereotaxic instrument. The following injection coordinates were used (mm from Bregma): anteroposterior (AP) 2.2, mediolateral (ML) ± 1.5, dorsoventral (DV) 1.8. Virus vectors were bilaterally injected at a flow rate of 0.1 μL/min and the total injection volume was 1 μL on each side. The frozen mouse brain tissue was cut into 12-μm thickness, and the virus infection efficiency was observed under a microscope (Olympus BX53, Japan) equipped with appropriate imaging technology.

### 2.12. Western blotting

Mice were sacrificed under urethane anesthesia and the hippocampus was dissected, quickly frozen in liquid nitrogen, and stored at -80°C for protein analysis. Western blotting was conducted following standard procedures. Briefly, hippocampal tissues or HT22 cells were lysed in ice-cold RIPA buffer (cat. no. R0010, Solarbio, Beijing, China). Equal amounts of protein were separated by 10% SDS–polyacrylamide gel electrophoresis and then transferred to PVDF membranes. The antibodies applied in this study were anti-BAIAP2 (cat. no. ab37542, Abcam, Cambridge, UK), anti-GluA1 (cat. no. ab183797, Abcam), anti-SYN 1 (cat. no. ab64581, Abcam), and anti-GAPDH (cat. no. AC033, ABclonal, Wuhan, China). The protein bands were developed using an enhanced chemiluminescent reagent and visualized employing a chemiluminescence detection system (Bio-Rad ChemiDoc touch imaging system, CA, USA).

### 2.13. Reverse transcription-quantitative PCR (RT-qPCR)

Total RNA isolated from the hippocampus was reverse transcribed into cDNA using the Revert Aid First Strand cDNA Synthesis Kit (cat. no. K1622, Thermo Scientific, Lithuania). qPCR was performed on a CFX96 Touch Deep Well Real-Time PCR Detection System (Bio-Rad, USA). Relative mRNA levels were calculated using the 2^–ΔΔCt^ method. The sequences of the primers used for qPCR are shown in Table 1.

**TABLE 1:** The primers used in the RT-qPCR.

Gene	Primer sequence
BAIAP2	Forward: 5′-GCTCAGGAGAATGTACCTGTCA-3′ Reverse: 5′-GGGTTGTAGTCCTCGCTG-3′
GAPDH	Forward: 5′-AGGTCGGTGTGAACGGATTTG-3′ Reverse: 5′-TGTAGACCATGTAGTTGAGGTCA-3′

### 2.14. Golgi staining

Brains were perfused sequentially with normal saline and 4% paraformaldehyde (PFA) solution, and then fixed in 4% PFA solution for 24 h before use. The brains were immersed in fixation solution from a Golgi staining kit (GMS 80020.1, GENMED, Shanghai, China) for 14 days. The brain was then immersed in 30% sucrose solution for 48 h and sliced into 100 μm-thick sections using a slicer (VT1200S, Leica, Germany). The sections were then placed on glass slides coated with gelatin and Golgi staining was performed according to the instructions of the Golgi staining kit. The stained sections were observed and imaged under a microscope (Olympus BX53, Japan).

### 2.15. Statistical analysis

All data were analyzed using SPSS software (version 22) and are expressed as means ± SEM. A two-tailed *t*-test was used for comparisons between two sets of data. A non-parametric test (Wilcoxon rank sum and signed rank tests) was used for comparisons between two sets of data when the assumption of homogeneity of variance was not met. One-way or two-way ANOVA followed by Tukey’s multiple comparison tests was used for comparisons among three or more groups. A *P*-value < 0.05 was considered significant.

## 3. Results

### 3.1. The expression levels of BAIAP2 were decreased in the hippocampi of mice exposed to CMS

**FIGURE 1:**
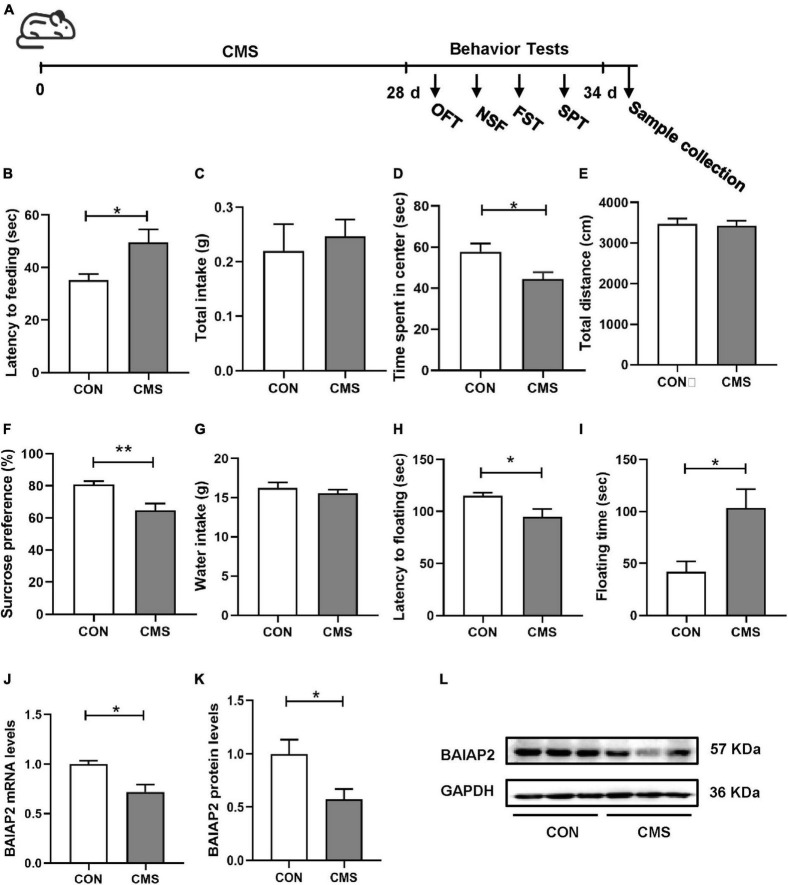
The expression levels of BAIAP2 were decreased in the hippocampi of mice exposed to chronic mild stress (CMS). **(A)** Timeline of CMS exposure and behavioral tests in mice. **(B)** Latency to feeding in the novelty-suppressed feeding (NSF) test. **(C)** Total food intake in the NSF test. **(D)** Time spent in the central area in the open field test (OFT). **(E)** Total distance traveled in the OFT. **(F)** Sucrose preference rate in the sucrose preference test (SPT). **(G)** Total water intake in the SPT. **(H)** Latency to floating in the forced swimming test (FST). **(I)** Floating time in the FST. **(J)** BAIAP2 mRNA expression as determined by RT-qPCR. **(K,L)** BAIAP2 protein expression as assessed by western blot. *n* = 10–15 per group for the behavioral tests, *n* = 5–6 per group for western blotting and RT-qPCR assays. *P < 0.05, **P < 0.01.

As shown in Figure 1A and Supplementary Table 1, mice were exposed to CMS for 4 weeks, following which depression- and anxiety-like behaviors were analyzed using four different behavioral assays. Compared with the control (CON) group, mice exposed to CMS exhibited longer latency to feeding (*t*_23_ = 2.204, *P* = 0.038; Figure 1B), showed no difference in food intake (*t*_23_ = 0.487, *P* = 0.631; Figure 1C) in the NSF test, spent less time in the center in the OFT (*t*_23_ = 2.540, *P* = 0.018; Figure 1D), and showed no difference in total distance traveled (*t*_23_ = 0.249, *P* = 0.677; Figure 1E) in the OFT. These results indicated that 4 weeks of exposure to CMS induced anxiety-like behaviors in mice.

In the SPT, the sucrose preference rate was lower in the CMS group than in the CON group (*t*_23_ = 2.961, *P* = 0.007; Figure 1F), whereas no difference in total water intake was observed among the groups (*t*_23_ = 0.869, *P* = 0.394; Figure 1G). In the FST, meanwhile, CMS-treated mice displayed shorter latency to first float (*Z* = −2.060, *P* = 0.039; Figure 1H) and longer floating time compared with mice in the CON group (*t*_23_ = 2.579, *P* = 0.017; Figure 1I). These results indicated that CMS induced depression-like behaviors in mice.

Relative to mice in the CON group, BAIAP2 mRNA and protein expression levels were significantly decreased in the hippocampi of CMS-treated mice as determined by RT-qPCR (*t*_7_ = 3.131, *P* = 0.017; Figure 1J) and western blotting (*t*_10_ = 2.614, *P* = 0.026; Figures 1K, L), respectively. These results suggested that BAIAP2 may be involved in regulating depression- and anxiety-like behaviors in mice submitted to CMS.

### 3.2. The expression levels of BAIAP2 were significantly decreased in CORT-treated HT22 cells

**FIGURE 2:**
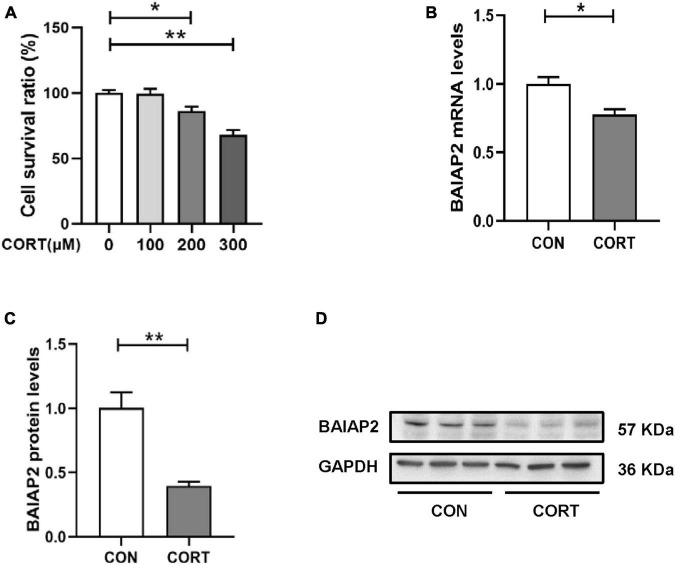
The expression levels of BAIAP2 were significantly decreased in corticosterone (CORT)-treated HT22 cells. **(A)** Cells were exposed to different CORT concentrations (0, 100, 200, and 300 μM) for 24 h and then assessed for viability using a Cell Counting Kit-8 (CCK-8) assay. **(B)** BAIAP2 mRNA and **(C,D)** protein expression levels 24 h after CORT treatment (200 μM) as assessed by RT-qPCR and western blot, respectively; *n* = 3–4 per group. *P < 0.05, **P < 0.01.

Chronic stress-induced depression- and anxiety-like behavior in mice is closely associated with elevated CORT levels *in vivo* (31, 32). Accordingly, CORT is frequently used to establish models of cellular stress *in vitro* (16, 33). Briefly, HT22 cells were treated with different concentrations of CORT (0, 100, 200 and 300 μM) for 24 h, and then the cell survival rate was measured by CCK-8 assay. No significant difference in cell viability was detected between the 0 and 100 μM CORT treatment groups, indicating that CORT did not affect cell viability when applied at physiological levels. However, at CORT concentrations of 200 μM and above, HT22 cell viability was markedly decreased [*F*_(3,12)_ = 0.344; *P* = 0.999, *P* = 0.047, and *P* < 0.001 for 100, 200 and 300 μM, respectively; Figure 2A]. These results indicated that high CORT concentrations significantly reduced HT22 cell viability in a dose-dependent manner. Next, the mRNA and protein expression levels of BAIAP2 in cells treated with 200 μM CORT were measured by qPCR and western blotting, respectively. The results showed that, compared with the CON (no CORT) condition, the expression of BAIAP2 was significantly reduced at both the mRNA (*t*_4_ = 3.541, *P* = 0.024; Figure 2B) and protein (*t*_4_ = 4.961, *P* = 0.008; Figures 2C, D) levels after treatment with 200 μM CORT for 24 h. These findings suggested that a link may exist between high CORT concentrations and the levels of BAIAP2.

### 3.3. BAIAP2 overexpression protected against CORT-induced cellular injury

**FIGURE 3:**
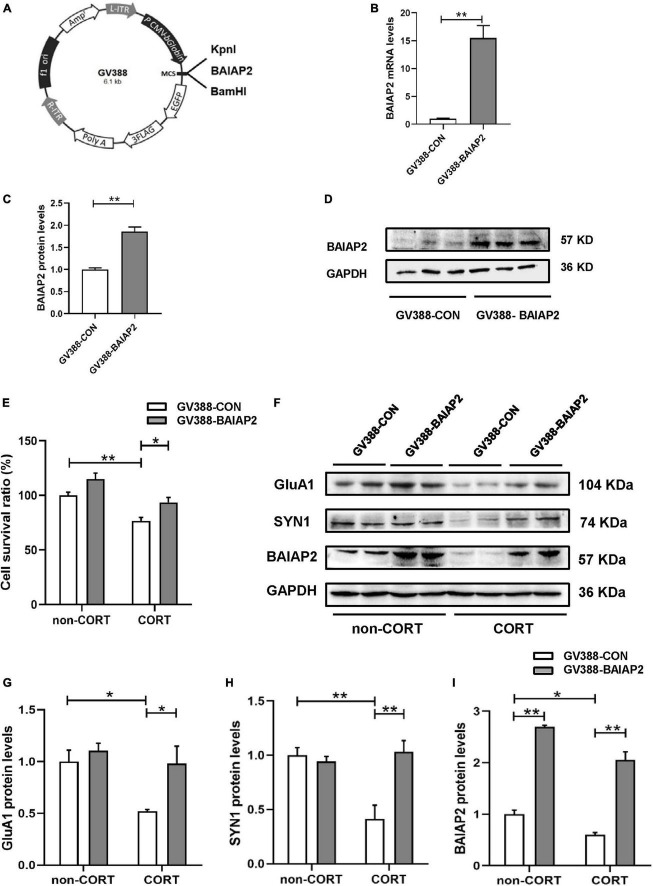
The overexpression of BAIAP2 improved the CORT-induced reduction in the viability of HT22 cells. **(A)** Schematic of the BAIAP2 overexpression plasmid. **(B)** BAIAP2 mRNA and **(C,D)** protein expression levels as determined by RT-qPCR and western blot, respectively. **(E)** Cell viability was assessed by Cell Counting Kit-8 (CCK-8) assay. **(F–I)** Western blot analysis of the expression of GluA1 **(F,G)**, SYN1 **(F,H)**, and BAIAP2 **(F,I)**; *n* = 3–4 per group. *P < 0.05, **P < 0.01.

A schematic of the construction of the BAIAP2 overexpression plasmid (GV388-BAIAP2) is shown in Figure 3A. HT22 cells were transfected with GV388-BAIAP2 and the expression efficiency was verified by RT-qPCR (*t*_4_ = 16.533, *P* = 0.003; Figure 3B) and western blotting (*t*_4_ = 7.799, *P* = 0.002; Figures 3C, D). To clarify whether BAIAP2 could prevent CORT-induced cell damage, HT22 cells were transfected with GV388-BAIAP2 and then treated with 200 μM CORT for 24 h. Two-way ANONA of the CCK-8 assay results showed that the survival of GV388-CON-transfected HT22 cells was significantly decreased with CORT treatment compared with that without; however, this effect was counteracted by BAIAP2 overexpression (GV388-BAIAP2) [*F*_(1, 16)_ = 0.070, *P* = 0.789; main effect of CORT: *F*_(1, 16)_ = 28.580, *P* < 0.001; main effect of BAIAP2: *F*_(1, 16)_ = 14.700, *P* = 0.002; Tukey’s *post-hoc* test: GV388-CON + non-CORT vs. GV388-CON + CORT, *P* = 0.005; GV388-CON + CORT vs. GV388-BAIAP2 + CORT, *P* = 0.046; Figure 3E]. These observations indicated that BAIAP2 overexpression significantly alleviated CORT-induced cell damage.

GluA1 is a key regulator of synaptic transmission and synaptic plasticity (34). Here, we found that overexpressing BAIAP2 in HT22 cells inhibited the CORT treatment-induced decrease in GluA1 protein expression [two-way ANOVA: *F*_(1, 12)_ = 2.776, *P* = 0.122; main effect of CORT: *F*_(1, 12)_ = 7.947, *P* = 0.016; main effect of BAIAP2: *F*_(1, 12)_ = 6.989, *P* = 0.021; Tukey’s *post-hoc* test: GV388-CON + non-CORT vs. GV388-CON + CORT, *P* = 0.032; GV388-CON + CORT vs. GV388-BAIAP2 + CORT, *P* = 0.044; Figures 3F, G]. Similar results were obtained for SYN1, a glycoprotein distributed in the membrane of presynaptic vesicles and is involved in the regulation of synaptic plasticity (35) [two-way ANOVA: *F*_(1, 12)_ = 13.700, *P* = 0.003; main effect of CORT: *F*_(1, 12)_ = 7.568, *P* = 0.018; main effect of BAIAP2: *F*_(1, 12)_ = 9.504, *P* = 0.010; Tukey’s *post-hoc* test: GV388-CON + non-CORT vs. GV388-CON + CORT, *P* = 0.003; GV388-CON + CORT vs. GV388-BAIAP2 + CORT, *P* = 0.002; Figures 3F, H]. BAIAP2 overexpression was also submitted to two-way ANOVA [*F*_(1, 12)_ = 1.173, *P* = 0.213; main effect of CORT: *F*_(1, 12)_ = 30.970, *P* < 0.001; main effect of BAIAP2: *F*_(1, 12)_ = 282.200, *P* < 0.001; Tukey’s *post-hoc* test: GV388-CON + non-CORT vs. GV388-CON + CORT, *P* = 0.047; GV388-CON + non-CORT vs. GV388-BAIAP2 + non-CORT, *P* < 0.001; GV388-CON + CORT vs. GV388-BAIAP2 + CORT, *P* < 0.001; Figures 3F, I]. These results suggested that the overexpression of BAIAP2 can prevent the CORT-induced decrease in the levels of synaptic plasticity-related proteins in HT22 cells, thereby enhancing the viability of the cells.

### 3.4. Hippocampal overexpression of BAIAP2 significantly alleviated CMS-induced behavioral abnormalities in mice

**FIGURE 4:**
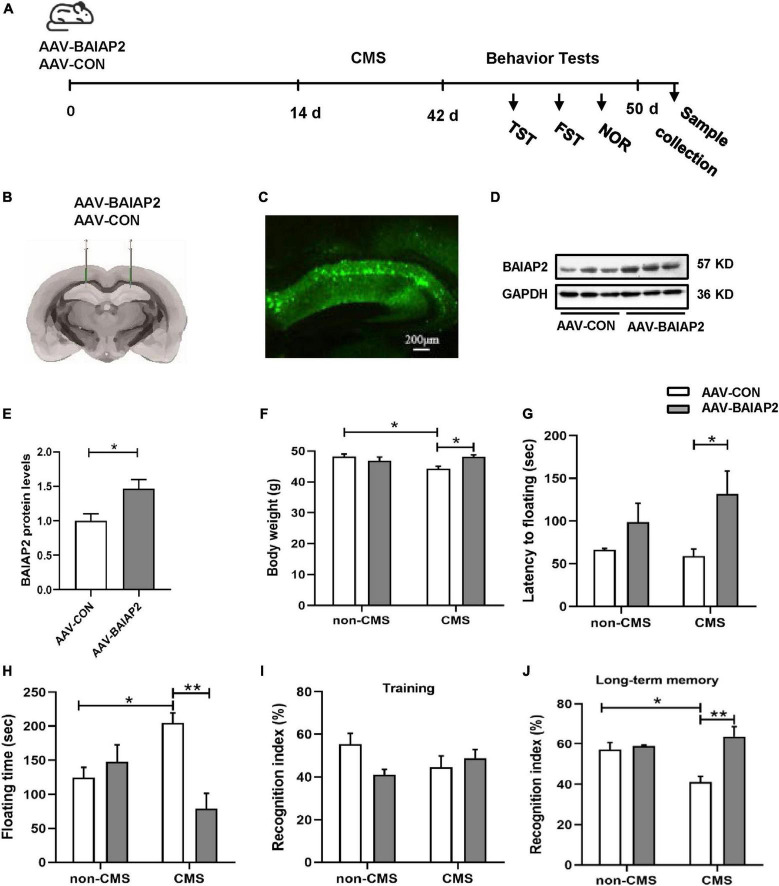
The overexpression of BAIAP2 in the hippocampus significantly alleviated behavioral abnormalities in mice exposed to chronic mild stress (CMS). **(A)** Timeline of CMS exposure and behavioral tests in mice. **(B,C)** The success of transfection was confirmed by the detection of fluorescence in the mouse hippocampus. **(D,E)** The overexpression of BAIAP2 in mice was confirmed by western blot. **(F)** Changes in body weight in the four groups of mice. **(G)** The latency to floating immobility and **(H)** floating immobility time of mice in the forced swimming test (FST). **(I)** The training stage and **(J)** the recognition index during the test stage in the novel object recognition (NOR) test. *n* = 8–10 per group for the behavioral tests; *n* = 6 per group for western blotting. *P < 0.05, **P < 0.01.

To further determine whether the overexpression of BAIAP2 could prevent CMS-induced depression-like behavior and concomitant cognitive impairment *in vivo*, bilateral hippocampal stereotactic injection of AAV-BAIAP2 or AAV-CON was performed once 2 weeks before the administration of CMS (Figure 4A). The success of AAV transfection was determined by fluorescence microscopic analysis of frozen mouse hippocampus sections (Figures 4B, C). BAIAP2 overexpression was confirmed by western blot analysis (*t*_10_ = 2.876, *P* = 0.017; Figures 4D, E).

Given that, in mice, the deletion of BAIAP2 and its re-expression after deletion has been shown to affect cognitive ability but not anxiety-like behavior (21, 36, 37), we next focused on the potential effects of BAIAP2 overexpression on memory and depression-like behavior in CMS-exposed mice. Two-way ANONA indicated that the overexpression of BAIAP2 significantly suppressed CMS-induced weight loss in mice [interaction *F*_(1, 32)_ = 9.329, *P* = 0.005; main effect of CMS: *F*_(1, 32)_ = 2.526, *P* = 0.122; main effect of BAIAP2: *F*_(1, 32)_ = 2.371, *P* = 0.133; Tukey’s *post-hoc* test: non-CMS + AAV-CON vs. CMS + AAV-CON, *P* = 0.016; CMS + AAV-CON vs. CMS + AAV-BAIAP2, *P* = 0.011; Figure 4F]. Compared with mice in the CMS + AAV-CON and non-CMS + AAV-CON groups, animals in the CMS + AAV-BAIAP2 group exhibited significantly longer latency to immobility [two-way ANOVA: interaction *F*_(1, 32)_ = 1.137, *P* = 0.294; main effect of CMS: *F*_(1, 32)_ = 0.462, *P* = 0.502; main effect of BAIAP2: *F*_(1, 32)_ = 7.669, *P* = 0.009; Tukey’s *post-hoc* test: CMS + AAV-CON vs. CMS + AAV-BAIAP2, *P* = 0.041; Figure 4G] and significantly shorter floating immobility time [interaction *F*_(1, 32)_ = 13.440, *P* = 0.001; main effect of CMS: *F*_(1, 32)_ = 0.008, *P* = 0.783; main effect of BAIAP2: *F*_(1, 32)_ = 6.410, *P* = 0.017; Tukey’s *post-hoc* test: non-CMS + AAV-CON vs. CMS + AAV-CON, *P* = 0.049; CMS + AAV-CON vs. CMS + AAV-BAIAP2, *P* = 0.001; Figure 4H] in the FST. These results suggested that the overexpression of BAIAP2 could alleviate the depression-like behavior induced by CMS.

In the NOR test, no differences in baseline recognition indices were observed among the four groups of mice during training [two-way ANOVA: interaction *F*_(1, 32)_ = 4.569, *P* = 0.040; main effect of CMS: *F*_(1, 32)_ = 0.133, *P* = 0.718; main effect of BAIAP2: *F*_(1, 32)_ = 1.329, *P* = 0.258; Figure 4I]. During the test period, mice in the CMS + AAV-BAIAP2 group (BAIAP2 overexpression) spent significantly more time exploring the new object as a percentage of total exploration time compared with that in the CMS + AAV-CON group [two-way ANONA: interaction *F*_(1, 32)_ = 8.905, *P* = 0.005; main effect of CMS: *F*_(1, 32)_ = 2.454, *P* = 0.127; main effect of BAIAP2: *F*_(1, 32)_ = 12.380, *P* = 0.001; Tukey’s *post-hoc* test: non-CMS + AAV-CON vs. CMS + AAV-CON, *P* = 0.019; CMS + AAV-CON vs. CMS + AAV-BAIAP2, *P* < 0.001; Figure 4J], suggesting that the overexpression of BAIAP2 in the hippocampus of mice could markedly inhibit CMS-induced cognitive impairment. Combined, these results demonstrated that overexpressing BAIAP2 in the hippocampus can prevent depression-like behavior and memory impairment induced by CMS in mice.

### 3.5. Hippocampal overexpression of BAIAP2 inhibited the CMS-induced decreases in the levels of synaptic plasticity-related proteins and dendritic spine density in mice exposed to CMS

**FIGURE 5:**
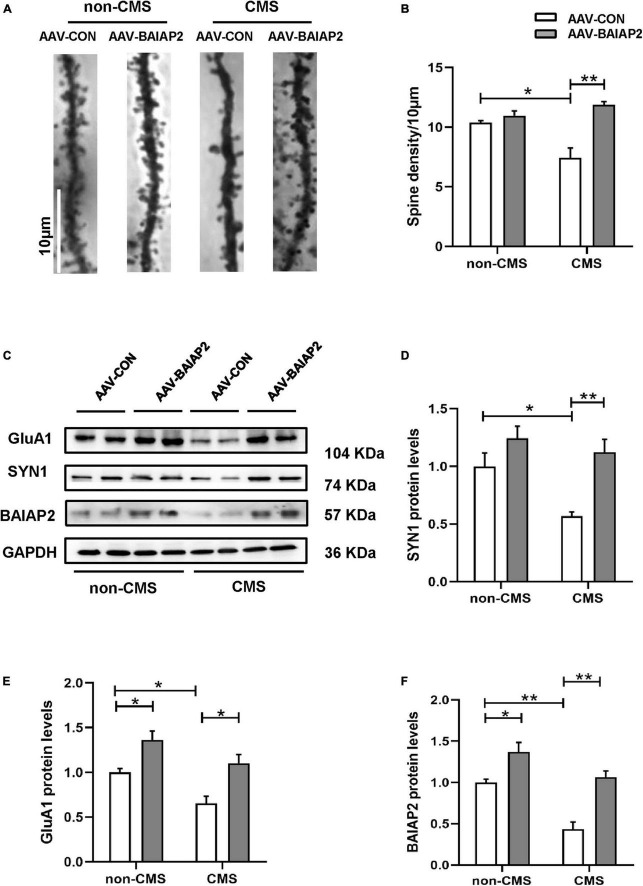
The overexpression of BAIAP2 in the hippocampus can inhibit the chronic mild stress (CMS)-induced decrease in the levels of synaptic plasticity-related proteins and dendritic spine density in mice. **(A,B)** Golgi staining showing dendritic spine density. **(C–F)** Western blotting analysis of the expression of SYN1 **(C,D)**, GluA1 **(C,E)**, and BAIAP2 **(C,F)**; *n* = 3–4 per group. *P < 0.05, **P < 0.01.

Next, we investigated the effects of BAIAP2 on structural synaptic plasticity by measuring dendritic spine density in hippocampal brain regions of mice using Golgi staining. The results showed that the overexpression of BAIAP2 reversed the CMS-induced decrease in dendritic spine density in mouse hippocampal neurons to a significant extent [two-way ANONA: interaction *F*_(1, 8)_ = 15.840, *P* = 0.004; main effect of CMS: *F*_(1, 8)_ = 4.432, *P* = 0.068; main effect of BAIAP2: *F*_(1, 8)_ = 27.100, *P* = 0.001; Tukey’s *post-hoc* test: non-CMS + AAV-CON vs. CMS + AAV-CON, *P* = 0.011; CMS + AAV-CON vs. CMS + AAV-BAIAP2, *P* = 0.001; Figures 5A, B].

Western blot analysis showed that the expression of the synaptic plasticity-related proteins SYN1 [two-way ANONA: interaction *F*_(1, 12)_ = 2.536, *P* = 0.137; main effect of CMS: *F*_(1, 12)_ = 7.969, *P* = 0.015; main effect of BAIAP2: *F*_(1, 12)_ = 16.570, *P* = 0.002; Tukey’s *post-hoc* test: non-CMS + AAV-CON vs. CMS + AAV-CON, *P* = 0.038; CMS + AAV-CON vs. CMS + AAV-BAIAP2, *P* = 0.008; Figures 5C, D] and GluA1 [interaction *F*_(1, 12)_ = 0.278, *P* = 0.608; main effect of CMS: *F*_(1, 12)_ = 13.750, *P* = 0.003; main effect of BAIAP2: *F*_(1, 12)_ = 24.620, *P* < 0.001; Tukey’s *post-hoc* test: non-CMS + AAV-CON vs. non-CMS + AAV-BAIAP2, *P* = 0.037; non-CMS + AAV-CON vs. CMS + AAV-CON, *P* = 0.048; CMS + AAV-CON vs. CMS + AAV-BAIAP2, *P* = 0.010; Figures 5C, E] was reduced in mice of the CMS + AAV-CON group; however, the decline in the expression of both proteins was greatly inhibited by BAIAP2 overexpression in the hippocampus of the mice [two-way ANOVA: interaction *F*_(1, 12)_ = 2.433, *P* = 0.145; main effect of CMS: *F*_(1, 12)_ = 26.870, *P* < 0.001; main effect of BAIAP2: *F*_(1, 12)_ = 35.710, *P* < 0.001; Tukey’s *post-hoc* test: non-CMS + AAV-CON vs. non-CMS + AAV-BAIAP2, *P* = 0.038; non-CMS + AAV-CON vs. CMS + AAV-CON, *P* = 0.002; CMS + AAV-CON vs. CMS + AAV-BAIAP2, *P* = 0.001; Figures 5C, F]. These results suggested that BAIAP2 overexpression prevented the CMS-induced decrease in dendritic spine density and levels of synaptic plasticity-related proteins in hippocampal neurons (Supplementary Figure 1).

## 4. Discussion

BAIAP2 is known to perform multiple and varied functions. For instance, BAIAP2 deficiency leads to abnormal renal tubulogenesis and altered tubular polarity and structure, supporting that BAIAP2 is crucial for ensuring the structural integrity and morphology of the plasma membrane (38). BAIAP2 also positively regulates the motility and invasive ability of fibrosarcoma cells, suggestive of its importance in the metastatic behavior of tumor cells (39). The deletion of BAIAP2 during embryonic development results in embryonic death at mid to late gestation, implying that BAIAP2-mediated pathways play a vital role in embryonic morphogenesis (40). In the central nervous system, meanwhile, BAIAP2 promotes postsynaptic density formation and is essential for synapse development and synaptic plasticity (41). BAIAP2 deficiency leads to defective actin/membrane regulation in dendritic spines, NMDA receptor dysfunction, and social and cognitive deficits (22, 42). BAIAP2 has also been linked to a variety of psychiatric disorders, including autism spectrum disorder (43), schizophrenia (44), and attention-deficit/hyperactivity disorder (21). Consistent with this, we found that BAIAP2 also plays a protective role against depression-like phenotypes in mice. The expression of BAIAP2 was downregulated in the hippocampus of CMS-exposed mice, while the upregulation of BAIAP2 prevented the depression- and anxiety-like behavior induced by CMS. Furthermore, the overexpression of BAIAP2 prevented CORT-induced HT22 cell injury *in vitro*, and inhibited the CMS-induced decrease in dendritic spine density and the downregulation of synaptic plasticity-related proteins in the hippocampus of mice *in vivo*.

Stress can lead to the loss of hippocampal excitatory synapses, dendritic atrophy, and the loss of dendritic spines (45), effects that are closely associated with stress-related diseases. Studies have shown that the levels of synaptic plasticity-related proteins and dendritic spine density are reduced in the hippocampus of patients with major depression (46). Consistent with these studies, we found that hippocampal dendritic spine density and synaptic plasticity-related protein concentrations were significantly decreased in mice submitted to CMS. The consequent downregulation of BAIAP2 in the hippocampus may have been closely related to the decreased dendritic spine density given that BAIAP2 is an essential regulator of dendritic spine dynamics and an important component of the postsynaptic density at excitatory synapses. To test this, we upregulated BAIAP2 expression in the hippocampus of mice and assessed the effect on dendritic spine density and depression- and anxiety-like behaviors. We found that overexpressing BAIAP2 in the hippocampus of CMS-treated mice not only alleviated the CMS-induced decrease in dendritic spine density but also largely prevented the consequent depression-like behavior and concomitant cognitive impairment. Changes at excitatory synapses underlie the basic pathophysiology of depression while reversing synaptic plasticity impairment is the key to alleviating depression-like symptoms (47). GluA1, a major component of AMPA receptors (AMPARs), mediates most of the fast excitatory synaptic transmission in the brain (34). SYN1, located in the presynaptic membrane, is an important regulator of synaptic plasticity (35). In the present study, we also found that the expression of GluA1 and SYN1 in the hippocampus of CMS-exposed mice was decreased and that this effect of CMS was inhibited by the overexpression of BAIAP2 in the hippocampus. These findings indicate that a strong association exists between the reversal of synaptic plasticity impairment and the anti-stress effect of BAIAP2.

Studies have shown that a sustained stress response activates the hypothalamic–pituitary–adrenal (HPA) axis, leading to the secretion of a large amount of CORT, which contributes to stress-induced depression-like behaviors (48). Indeed, increased CORT levels *in vivo* can induce depression-like behaviors in mice (48, 49). We found that the expression levels of BAIAP2 were significantly decreased in CORT-treated HT22 cells, which was consistent with our *in vivo* analysis in mice. To test whether BAIAP2 has neuroprotective effects, we overexpressed BAIAP2 in HT22 cells and found that it had no effect on cell survival in the absence of CORT treatment; however, the overexpression of BAIAP2 improved the CORT-mediated decrease in cell viability and expression of GluA1 and SYN1. These results highlighted the fundamental role of BAIAP2 in the regulation of stress. It is known that siRNA-mediated knockdown of BAIAP2 reduces the density, length, and width of neuronal dendritic spines and alters synaptic transmission (36, 50). Although we demonstrated that the overexpression of BAIAP2 produces antidepressant effects, one limitation of the present study was that we did not inhibit the activity or the expression of BAIAP2. Despite this, BAIAP2 remains a potential therapeutic target for depression. Notably, in addition to the brain, BAIAP2 is also expressed in epithelial tissues, including vascular epithelial cells, and it is now known that people with depression are at an increased risk of cardiovascular disease (51). Whether BAIAP2 secreted by vascular endothelial cells acts on neurons, thereby exerting an antidepressant effect, merits further investigation.

In conclusion, in the present study, we demonstrated that BAIAP2 can prevent stress-induced depression-like behaviors and cognitive impairment in mice, strongly suggesting its potential in the treatment of depression and perhaps other stress-induced psychiatric disorders.

## Data availability statement

The raw data supporting the conclusions of this article will be made available by the authors, without undue reservation.

## Ethics statement

The animal study was reviewed and approved by the Medical Ethics Committee of Hebei Medical University.

## Author contributions

HS and YS conceived and managed the study. YF, HS, and YS designed the study. YF, XG, RY, HF, XY, and ShuW performed the experiments. YF, XG, and XW analyzed the data and prepared the figures. YF and PZ wrote the manuscript with input from HS, HF, LS, RY, SheW, ShuW, XW, XG, XY, and YS. LS and SheW improved the experimental scheme. All authors reviewed and approved the submitted version of the manuscript.
