# Supplementary material for: Hippocampal BAIAP2 prevents chronic mild stress-induced depression-like behaviors in mice
Source: Front Psychiatry. 2023 May 10;14:1192379. doi: 10.3389/fpsyt.2023.1192379 (PMC10206043; doi:10.3389/fpsyt.2023.1192379)
Supplement: Supplementary file 1 [file Image_1.pdf]

Supplementary Figures

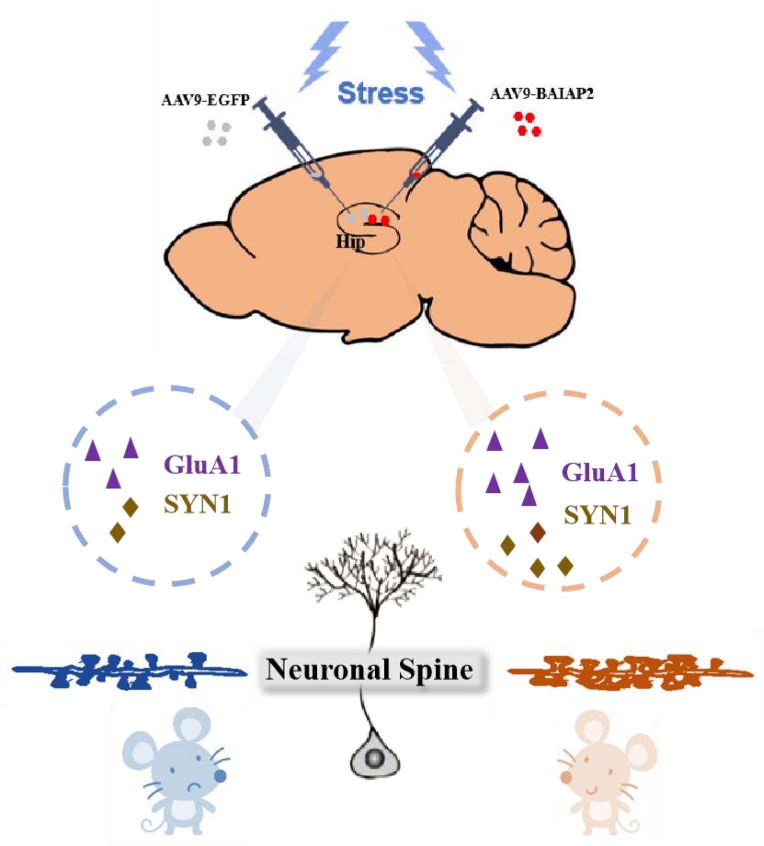

Figure 1. The graphic abstract. Overexpression of BAIAP2 in the hippocampus reverses chronic mild stress-induced changes in synaptic plasticity-related proteins GluA1 and SYN1, thereby improving dendritic spine density and alleviating depressive and anxiety-like behavior in mice.
