# Supplementary material for: Hippocampal BAIAP2 prevents chronic mild stress-induced depression-like behaviors in mice
Source: Front Psychiatry. 2023 May 10;14:1192379. doi: 10.3389/fpsyt.2023.1192379 (PMC10206043; doi:10.3389/fpsyt.2023.1192379)
Supplement: Supplementary file 2 [file Table_1.DOCX]

Supplementary Table

|  | Stressor-1 | Stressor-2 |
| --- | --- | --- |
| Day1 | crowding | light/dark cycle reversal |
| Day2 | food deprivation | cage tilting and empty |
| Day3 | water deprivation | restraint |
| Day4 | tail clamp | damp bedding |
| Day5 | crowding | light/dark cycle reversal |
| Day6 | cage tilting and empty | food deprivation |
| Day7 | water deprivation | tailing clamp |
| Day8 | Restraint | cold stimulation |
| Day9 | cage tilting and empty | water deprivation |
| Day10 | food deprivation | tailing clamp |
| Day11 | crowding | damp bedding |
| Day12 | water deprivation | restraint |
| Day13 | cold stimulation | damp bedding |
| Day14 | crowding | tailing clamp |
| Day15 | food deprivation | crowding |
| Day16 | cage tilting | water deprivation |
| Day17 | light/dark cycle reversal | restraint |
| Day18 | food deprivation | damp bedding |
| Day19 | crowding | restraint |
| Day20 | cage tilting | damp bedding |
| Day21 | cold stimulation | water deprivation |
| Day22 | food deprivation | restraint |
| Day23 | crowding | tailing clamp |
| Day24 | cage tilting and empty | light/dark cycle reversal |
| Day25 | restraint | cold stimulation |
| Day26 | food deprivation | damp bedding |
| Day27 | crowding | tail clamp |
| Day28 | cage tilting | damp bedding |

Supplementary Table 1. The CMS protocol
